# Evaluation of a Single Knee System: All-Polyethylene Tibia (APT) vs. Metal-Backed Tibia (MBT) in Primary Total Knee Arthroplasty

**DOI:** 10.7759/cureus.94155

**Published:** 2025-10-08

**Authors:** Benjamin Huang, Joshua Vanhoozier, Charlotte Huang, Russell T Nevins

**Affiliations:** 1 College of Osteopathic Medicine, Touro University Nevada, Henderson, USA; 2 Statistics, University of California Santa Barbara, Santa Barbara, USA; 3 Orthopaedics, Valley Health System, Las Vegas, USA

**Keywords:** all polyethylene tibial component, nrs pain score, propensity score matching (psm), retrospective cohort, tka

## Abstract

Background

The all-polyethylene tibia (APT) in total knee arthroplasty (TKA) previously demonstrated similar survival and function compared to metal-backed tibia (MBT), without the associated risk of backside wear and at a lower cost. The LinkSymphoKnee (LSK) (Waldemar Link GmbH & Co. KG, Hamburg, Germany) and its corresponding APT received FDA 510(k) approval in 2021; however, clinical outcome data remain limited. The purpose of this study was to determine whether the LSK APT would have non-inferior pain outcomes compared to the LSK MBT within two years postoperatively and compile descriptive statistics on revision, repeat surgery, manipulation under anesthesia, and range of motion.

Methods

This retrospective cohort study analyzed data from patients who underwent primary TKA performed by a single surgeon at a single center between January 2021 and October 2024. Patient chart data were categorized based on tibial implant design(APT vs MBT). After applying exclusion criteria, propensity score matching incompletely but significantly reduced baseline differences between groups. A linear mixed-effects model was employed to analyze differences in pain with non-inferiority of APT for pain reduction, tested against a 10% delta. The study also compiled statistics on implant survival and adverse events.

Results

After best-case propensity score matching, 229 patients were included in each group (APT and MBT). The APT group was significantly older (mean 77.0 years vs. 71.0 years, p < 0.001), had a lower BMI (mean 28.9 vs. 30.9, p < 0.001), and contained more females (65 vs 92, p<0.008) compared to the MBT group. Numerical Rating Scale (NRS) pain scores decreased over time in both groups. The 95% confidence interval in NRS pain scores was (-0.51, 0.19), indicating no statistically significant difference in scores. Flexion range of motion was similar between groups, reaching approximately 120 degrees. The extension range of motion was also comparable, near zero degrees. Three patients with APT and one patient with MBT required manipulation under anesthesia within eight weeks. One revision of the femur occurred in the APT group, while two full revisions occurred in the MBT group. Additional adverse events included synovectomy, patella fracture, and polyethylene insert exchange.

Conclusion

This study suggests that APT in TKA demonstrates non-inferior pain outcomes compared to MBT when performed by a fellowship-trained surgeon using the LSK system. While descriptive analysis showed some differences in other outcomes like revision and manipulation under anesthesia, further research with larger, randomized controlled trials is needed to confirm these findings and assess long-term functional outcomes and patient satisfaction.

## Introduction

Total knee arthroplasty (TKA) is a definitive treatment for advanced osteoarthritis, providing substantial pain relief and functional improvement. The demand for TKA is expected to rise significantly, with projections reaching 3.05 million procedures by 2060, driven by an aging population and increasing rates of obesity [1]. Implant selection is a critical aspect of TKA, influenced by patient-specific factors, surgeon preferences, and cost considerations [2].

The all-polyethylene tibia (APT) represents a cost-effective alternative to the modular metal-backed tibia (MBT), especially in developing countries [3-5]. The APT utilizes a monoblock design that eliminates the potential for backside wear, a known complication in modular implants [6]. Historical studies on APT showed limited indications due to poor survivorship in young, more active patients [7,8]. Developments such as cross-linking, additives, and improved geometry with coronal stability have markedly improved clinical outcomes [9,10]. 

Recent studies have shown comparable clinical outcomes between APT and MBT in TKA [11]. Notably, a recent analysis of the American Joint Replacement Registry reported a lower risk of revision TKA with APT [12]. The LinkSymphoKnee (LSK) system from Waldemar Link GmbH & Co. KG (Hamburg, Germany) received FDA approval in 2021, and specific data on the APT component’s clinical outcomes remain limited. Therefore, a foundational study is essential to document the clinical outcomes of this specific implant, particularly in direct comparison to the LSK MBT, which shares an identical geometry. Pain reduction, survival, and incidence of adverse events are considered useful preliminary data to capture for patient safety and to justify the cost savings associated with the APT design.

This study aimed to investigate the non-inferiority of the APT compared to MBT components in the LSK system through a retrospective chart analysis. The primary objective was to assess differences in pain reduction between the two groups. Secondary objectives included the evaluation of implant survival, the rate of manipulation under anesthesia, and other adverse events. Using a predefined delta of 10%, APT has non-inferior pain outcomes if the upper bound of the confidence interval for the difference in numerical rating scale (NRS) pain scores is no more than one point worse than the MBT group. This delta was chosen through the clinical expertise of the surgeon in this study (RN).

## Materials and methods

This retrospective cohort study examined patient charts from a single surgeon between January 2021 and October 2024. The Institutional Review Board (IRB) waived the requirement for approval, as this research was not considered human subjects research and the data collected was part of routine medical care (approval number TUNIRB000323). Data collection was performed using a unique identifying number, and no identifiable information was collected. The study design was finalized before any statistical analysis was performed.

The surgery was performed through a median parapatellar incision, and mechanical alignment was used with traditional posterior referencing. Patient charts were filtered using the International Classification of Diseases (ICD)-10 code Z95.65. Exclusion criteria included revision TKA, constrained implants (stems, constrained condylar knee (CCK), hinge), crucial-retaining designs, use of alternative bearing surfaces (ultra-congruent (UC) polyethylene inserts), or concomitant procedures (e.g., TKA with open reduction and internal fixation (ORIF)). Indications for APT included patients aged over 70 years and those with a BMI less than 38. 

Before data collection, a power analysis was performed using a sample size calculator. Previously published data taken from a similar patient population in the early postoperative period (0-3 months) [13] were used to calculate the required sample size that could detect a 10% difference in outcomes: at a .95 significance level and a power of 0.8, 175 patients per group was needed.

Data were extracted from the electronic medical record (EMR) and recorded in a password-protected spreadsheet on secure computers, accessible only to the principal investigator (PI) and designated data abstractors. To ensure data accuracy, a randomly selected subset of 10% of charts was reviewed by two abstractors, and inter-rater reliability was assessed using Cohen's kappa. Adjustments were made to the data abstraction form to improve workflow and reduce error rates. Data collected included: date of birth, date of surgery, preoperative pain, follow-up dates, pain scores at each follow-up, range of motion (ROM) at each follow-up, body mass index (BMI), gender, smoking status, and tibial implant design. Patient names were not recorded; medical record numbers (MRNs) were used for internal tracking. The de-identified dataset was accessible to the data abstractors, PI, and data analyst.

Pain scores were assessed using the Numerical Rating Scale (NRS) from 0 to 10. Range of motion was measured in degrees of flexion and extension. Implant survival was defined as the absence of any revision surgery for any reason. Adverse events, such as the need for manipulation under anesthesia, other surgery, or falls, were also documented. 

Statistical analysis was performed using R software, version 4.4.2 (R Foundation for Statistical Computing, Vienna, Austria). To minimize confounding due to baseline differences between the groups (APT vs MBT), propensity score matching was employed. A logistic regression model was used to calculate propensity scores based on BMI, age, gender, and smoking status. A 1:1 matching algorithm was then used, and any demographic differences were determined. A linear mixed-effects model was used to analyze differences in NRS pain scores over time, accounting for within-patient correlations. Descriptive statistics were compiled for flexion and extension, manipulation under anesthesia, and other adverse events.

Non-inferiority was assessed by defining a delta of 10% worse reduction in pain for the APT group compared to the MBT group. On the NRS pain scale, this translated to a one-point difference in average score. The confidence intervals of the difference in pain reduction between the two groups were then examined to determine if the lower bound of the confidence interval was greater than this one-point non-inferiority margin.

## Results

Demographic data

A total of 653 patient records satisfied the implant type requirement. After 1:1 propensity score matching, 229 patients were included in each group (APT and MBT), yielding a total matched cohort of 458 patients. At the most recent visit, the average follow-up period for the APT group was 201±196 days (range: 11-1116). For the MBT group, the average follow-up period was 192±186 days (range: 10-999). 

The APT group had a lower proportion of males (65 patients, 28%) compared to the MBT group (92 patients, 40%, p=0.008). The mean age in the APT group was 77.0±5.6 years, and in the MBT group, it was 71.0±5.2 years. The mean BMI in the APT group was 28.9±4.7lb/in^2^, and in the MBT group, it was 30.9±5.4lb/in^2^. Laterality and smoking status showed no significant differences using Chi-square analysis. After data collection was completed, the data underwent a verification process. At each follow-up, the number of days since surgery was calculated, and any negative or anomalous values were verified. This process was performed for a total of 12 charts. Cohen’s kappa score was 0.92. Demographic data are shown in Table 1.

**Table 1:** Demographic Data APT: All-polyethylene tibia, MBT: Metal-backed tibia. p<.05 was considered significant, marked with a *

Characteristic	APT Group (n=229)	MBT Group (n=229)	Statistical Test (Value, df)	p-value
Age, mean (SD), years	77.0 (± 5.6)	71.0 (± 5.2)	Independent t-test	<0.001*
BMI, mean (SD), kg/m^2	28.9 (± 4.7)	30.9 (± 5.4)	Independent t-test	<0.001*
Males, n (%)	65 (28%)	92 (40%)	χ2 (7.06, 1)	0.008*
Left knee, n (%)	115 (50.2%)	112 (49.1%)	χ2 (0.08, 1)	0.78
Smoking status, n (%)	-	-	χ2 (1.26, 3)	0.74
Current smoker, n (%)	13 (5.7%)	18 (7.9%)	-	-
Previous smoker, n (%)	68 (29.7%)	61 (26.6%)	-	-
Never smoker, n (%)	136 (59.4%)	139 (60.7%)	-	-
Smoking status not specified, n (%)	12 (5.2%)	11 (4.8%)	-	-

**Figure 1:**
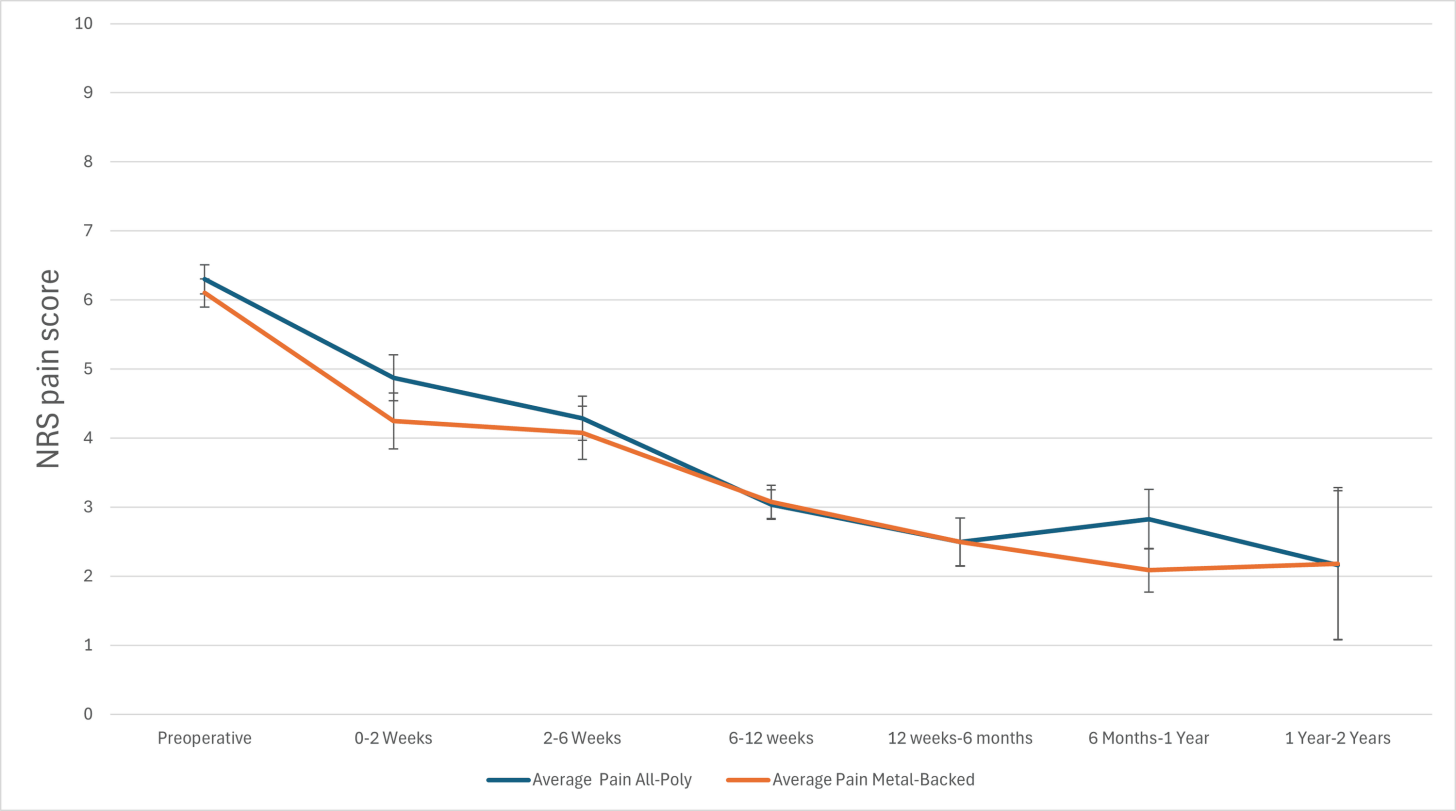
APT vs MBT postoperative pain APT: All-polyethylene tibia, MBT: Metal-backed tibia.

Numerical rating scale (NRS) pain scores decreased over time in both groups following TKA, as shown in Figure 1. Mean NRS pain scores for the MBT group were: 4.2 (± 2.7) at 0-2 weeks, 4.1 (± 2.9) at 2-6 weeks, 3.1 (± 2.4) at 6-12 weeks, 2.5 (± 2.7) at 3-6 months, 2.1 (± 2.4) at 6-12 months, and 2.2 (± 2.2) at 1-2 years. For the APT group, mean scores were: 4.9 (± 2.7), 4.3 (± 2.3), 3.0 (± 2.4), 2.5 (± 2.5), 2.8 (± 2.8), and 2.2 (± 3.0) for the respective time bins. Preoperatively, the mean NRS pain score for the APT group was 6.3 (± 2.5), and for the MBT group, it was 6.1 (± 2.5).

The linear mixed-effects model estimated the difference between NRS pain scores (APT minus MBT), resulting in a 95% confidence interval of (-0.51, 0.19). As the upper bound of the confidence interval is less than 1.0, APT demonstrated non-inferior outcomes compared to MBT. NRS pain scores decreased over time in both groups following TKA, as shown in Figure 1. Mean NRS pain scores for the MBT group were: 4.2 (± 2.7) at 0-2 weeks, 4.1 (± 2.9) at 2-6 weeks, 3.1 (± 2.4) at 6-12 weeks, 2.5 (± 2.7) at 3-6 months, 2.1 (± 2.4) at 6-12 months, and 2.2 (± 2.2) at 1-2 years. For the APT group, mean scores were: 4.9 (± 2.7), 4.3 (± 2.3), 3.0 (± 2.4), 2.5 (± 2.5), 2.8 (± 2.8), and 2.2 (± 3.0) for the respective time bins. Preoperatively, the mean NRS pain score for the APT group was 6.3 (± 2.5), and for the MBT group, it was 6.1 (± 2.5). The linear mixed-effects model estimated the difference between NRS pain scores (APT minus MBT), resulting in a 95% confidence interval of (-0.51,0.19). As the upper bound of the confidence interval is less than 1.0, APT demonstrated non-inferior outcomes compared to MBT. Pain scores are shown in Figure 1.

**Figure 2:**
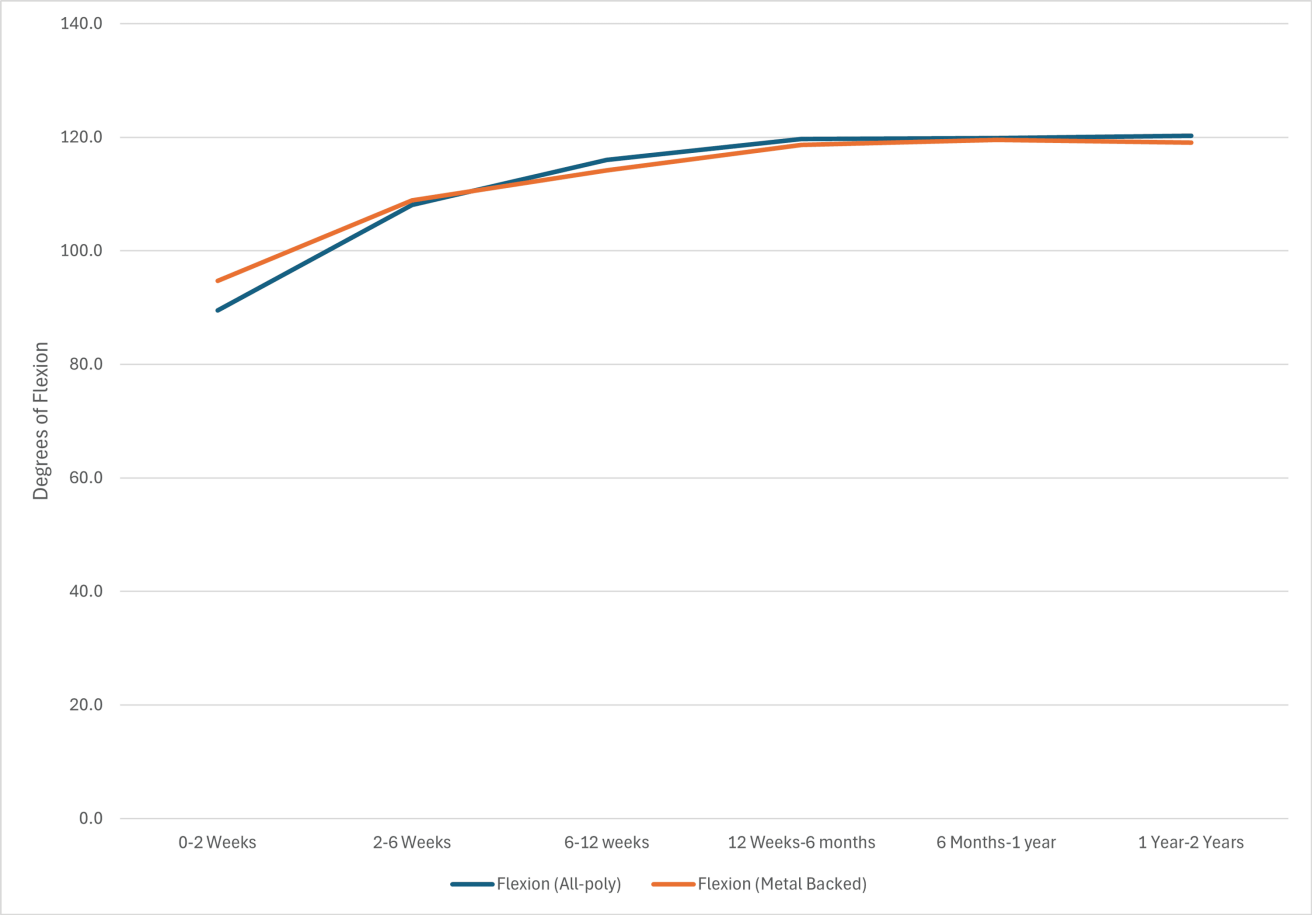
APT vs MBT flexion APT: All-polyethylene tibia, MBT: Metal-backed tibia.

Flexion and extension range of motion (ROM) were evaluated for both groups across the follow-up period. Mean flexion ROM for the APT group was: 89.5 (± 15.7), 108.1 (± 13.7), 116.0 (± 10.2), 119.7 (± 4.31), 119.9 (± 3.3), and 120.3 (± 1.6), respectively. For the MBT group, mean flexion ROM was: 94.7 (± 13.7), 108.9 (± 18.4), 114.2 (± 12.9), 118.7 (± 7.2), 119.5 (± 5.1), and 119.0 (± 7.2), respectively. Flexion range of motion in the APT and MBT groups is shown in Figure 2.

**Figure 3:**
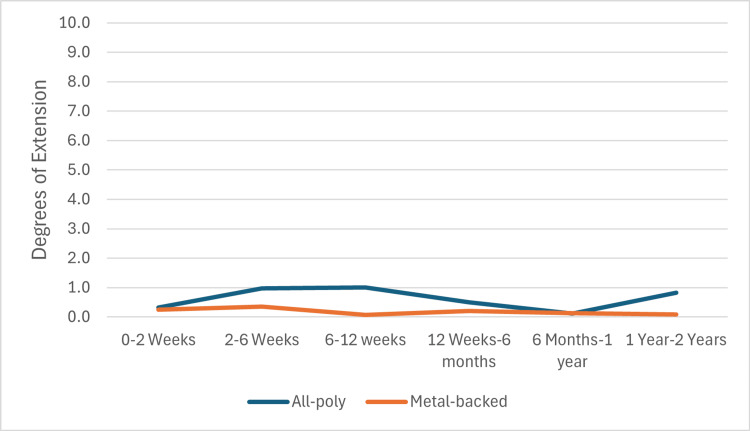
APT vs MBT extension APT: All-polyethylene tibia, MBT: Metal-backed tibia.

Mean extension ROM for the APT group was: 0.3 (± 2.8), 1.0 (± 2.9), 1.0 (± 2.9), 0.5 (± 3.2), 0.1 (± 2.1), and 0.8 (± 5.9), respectively. For the MBT group, mean extension ROM was: 0.3 (± [SD]), 0.3 (± 2.2), 0.1 (± 2.6), 0.2 (± 2.8), 0.1 (± 2.8), and 0.1 (± 1.3), respectively. No clinically meaningful differences were observed in either flexion or extension ROM between the two implant groups. The extension range of motion in the APT and MBT groups is shown in Figure 3.

The indication for manipulation under anesthesia is the inability to achieve less than ninety degrees of flexion at six weeks post-surgery. Three patients in the APT group and one in the MBT group required manipulation under anesthesia. Implant survival in the APT group included one revision of the femoral component at 364 days post-surgery. In the MBT group, two patients required revision at 67 and 1052 days post-surgery. Other adverse events were also documented. A left knee anterior arthrotomy and synovectomy was performed at 620 days following APT TKA due to synovitis. An MBT TKA patient underwent synovectomy and polyethylene insert exchange at 498 days status post-TKA due to patellar clunk syndrome.

## Discussion

This retrospective chart review analysis aimed to compare early pain outcomes between APT and MBT components in TKA. Our primary finding indicates that the APT is non-inferior to MBT within two years postoperatively. This finding aligns with other studies reporting comparable functional outcomes, pain scores, and patient satisfaction with APT [11,12]. By focusing on early pain trajectories and complications, this study supports existing literature, particularly given that early pain and complications are predictive of long-term functional outcomes [14,15].

For surgeons operating in high-volume inpatient settings, this study supports the use of APT using the indications of age over 70 years and BMI less than 38. The LSK system has identical geometry for both APT and MBT components, and these good outcomes are likely due to material differences. The ideal direction for future research would be a blinded randomized controlled trial comparing the LSK MBT TKA with the respective APT. This would include validated knee function scores, such as the Knee injury and Osteoarthritis Outcome Score (KOOS) or the Western Ontario and McMaster Universities Osteoarthritis Index (WOMAC).

This study was not adequately powered to detect differences in implant survival or the incidence of manipulation under anesthesia over extended periods. Descriptively, neither group showed obvious differences that suggest early failure of the APT. The APT group experienced one revision (of the femoral component due to severe flexion contracture), while two patients in the MBT group required revision, with no revisions in either group attributed to septic or aseptic loosening of the tibial component. Secondary outcome measures, such as range of motion, showed no clinically meaningful differences. While the APT group had 0.4 fewer degrees of extension, this difference is not clinically relevant.

This study has several limitations that warrant consideration. First, as a retrospective chart review, it is susceptible to inherent biases, and data collection relies on the completeness and accuracy of medical record documentation. Propensity score matching was utilized in a 1:1 ratio, aiming to achieve caliper-matched cohorts from the MBT group. While baseline differences were minimized, residual differences persisted in age (77 vs. 71 years), BMI (28.9 vs. 30.9), and gender (28% vs. 40% male) between the APT and MBT groups. These baseline imbalances, which have been associated with worse outcomes in TKA [7,8,16], could potentially confound the results. However, this analysis demonstrated non-inferiority for pain outcomes, suggesting that the baseline imbalances did not negate the comparable pain trajectories observed.

Second, the relatively short average follow-up time, at 201 days for the APT group and 192 days for the MBT group, limits the applicability of these results to the short term. While the linear mixed-effects model accounted for patients with less data, the robustness of these findings is limited in the longer follow-up periods. 

Third, the study's data were collected from a single surgeon at a single center within a large private practice, which introduces a risk of clustering at the provider and institutional levels. This may limit the external validity and generalizability of the findings to other surgical practices or patient populations. The substantial proportion of APT TKAs (33% of the original dataset) performed by this surgeon suggests a potential "learning effect" related to the unique surgical technique required for APT due to its lack of modularity. This could have refined the surgeon's technique over time, potentially leading to improved outcomes that might not be replicated by surgeons who do not routinely use APT TKA [17].

## Conclusions

This analysis suggests that the LSK APT has non-inferior pain outcomes compared to MBT in an inpatient setting, done by a joint replacement surgeon. The risk of other events, such as revision and manipulation under anesthesia, shows differences, although no statistical analysis was performed. Future studies could be done in a large sample randomized controlled trial in long-term clinical outcomes, with outcomes focused on validated function scores and patient satisfaction.
